# The Theater as a Disease-Spreader

**Published:** 1905-09

**Authors:** 


### The Theater as a Disease-Spreader.

DR. S. H. BROWN in a recent paper on the theatrical profession and disease, said he considered the profession is a factor in the dissemination of parasitic diseases, and concludes as follows:

1. That the theatrical profession is a factor of great importance in the dissemination of scabies, pediculosis, syphilis, and gonorrhea, by reason of its peculiar sociological position.
2. That this influence is greater in traveling troupes than in companies located permanently in the large cities on account of the lack of accommodations and unhygienic conditions under which they live.
3. Living apart from the rest of the world as they do to a great extent they are deprived of the medical education which is becoming so popular with the rest of the laity.
4. Lack of time prevents the members of this profession from consulting able medical men in the large cities except when physically incapacitated, and in consequence they fall into the hands of unscrupulous and ignorant physicians or charlatans.

This is not only unjust to a large body of men and women, but it is distinctly untrue. The theatrical profession is not, as one would infer from Brown's conclusions a profession of pediculi-infected prostitutes; nor is it any more uncleanly than any other class of people. On the contrary, as a profession, it is distinctly cleanly. So far as hygienic surroundings are concerned the statements are in a measure true, but this condition of affairs is not the fault of the individual members of the profession. The fault lies at the doors of local theater managers and health commissioners who allow the maintenance of filthy and illy-ventilated dressing rooms with unsanitary surroundings. The exigencies of the life of an actor make unhealthy living almost a matter of necessity. They get little sleep as a rule, especially when they are making a series of "one-night stands"; they eat in railroad restaurants, a great many of them at boarding houses and small hotels; their meal hours are irregular; but they are cleanly. It is quite likely that Brown has looked at the profession from in front of the footlights and gazed at them through the eyes of that class of sodden purists who cannot see anything but evil in the theater and moral putrescence in all those who have any connection with it.

The other counts in the Brown indictment are equally fallacious. The theatrical profession is medically educated; it has to be for self-protection, although it may be granted that it is equally as careless of its individual self in hygienic matters as most people outside the ranks of the profession. Actors are medically educated, for on their good health depends their livelihood; they are careful of their physical well-being; and on the authority of a Buffalo physician who for years has been intimately associated with the actors in a professional capacity, they are better and more conscientious patients than the average run of people. Every city in which theaters are maintained has one or more physicians who are known as "theatrical doctors." Any one of these men will bear witness to the statement that during the theatrical season they are busy keeping actors in good physical condition. It is true that in a measure the actor lives in a world of his own, a world wherein he finds congeniality and sympathy; in society he is rather looked upon as a detached creature; yet with a more careful study of the question Brown might have reached these truthful conclusions and added them to his own unfair opinions—namely, that the theatrical profession, in the large majority of cases, does not give, but receives venereal disease from society; that the actor is an honest patient, rigidly following instructions and, for the most part, never failing to pay his bills.

